# Protective Effect of Maritime Quarantine in South Pacific Jurisdictions, 1918–19 Influenza Pandemic

**DOI:** 10.3201/eid1403.07-0927

**Published:** 2008-03

**Authors:** Melissa A. McLeod, Michael Baker, Nick Wilson, Heath Kelly, Tom Kiedrzynski, Jacob L. Kool

**Affiliations:** * University of Otago, Wellington, New Zealand; † Victorian Infectious Diseases Reference Laboratory, Melbourne, Australia; ‡ Secretariat of the Pacific Community, Noumea, New Caledonia; § World Health Organization Office for the South Pacific, Suva, Fiji

**Keywords:** pandemic influenza, quarantine, border control, South Pacific jurisdictions, dispatch

## Abstract

We reviewed mortality data of the 1918–19 influenza pandemic for 11 South Pacific Island jurisdictions. Four of these appear to have successfully delayed or excluded the arrival of pandemic influenza by imposing strict maritime quarantine. They also experienced lower excess death rates than the other jurisdictions that did not apply quarantine measures.

Recent pandemic plan development by many countries suggests the international concern about pandemic influenza (*1*). However, no work has been published to date to inform such planning by evaluating islands’ border control practices to prevent the arrival of pandemic influenza. Yet border control is potentially easier to study for islands than for states with porous land borders, and for many island states with limited health and economic resources border control may provide the only practical defense against the introduction of pandemic influenza.

## The Study

We aimed to identify the features that distinguished successful from unsuccessful border control attempts to exclude pandemic influenza from South Pacific Island jurisdictions (including the “continental” island of Australia) during the 1918–19 influenza pandemic. Jurisdictions were defined as countries, territories, or states within federal systems that had the capacity to implement their own border control measures. Although island jurisdictions in the Pacific are widely dispersed geographically, it appears that nearly all were at some risk for the spread of pandemic influenza from ship-borne contact. The details of ship-borne spread of this pandemic in the Pacific have been well documented (*2*,*3*). Indeed, we have only been able to identify 1 area in the South Pacific that had no reported arrival of the pandemic in the 1918–1922 period, i.e., the geographically remote Lau and Yasawa Islands (in the Fiji Group) (*2*).

Data on quarantine, pandemic arrival, and pandemic-attributable health effects were accessed through a systematic search of Medline, Embase, Australasian Medical Index, and Web of Science. Archival data were accessed directly from the National Archives (in Wellington, New Zealand, and Canberra, Australia) and from government departments and websites for New Zealand; Australia; the Secretariat for the Pacific Community Headquarters in Noumea, New Caledonia; and the World Health Organization.

Our literature search identified 35 articles and documents that included information on the use of border control in 11 of 25 South Pacific Island jurisdictions. An additional 21 archival documents were reviewed. Four jurisdictions in this region met our definition of strict maritime quarantine (monitoring all passengers and crew for at least 1 day before disembarking was permitted). These jurisdictions were American Samoa (5 days’ quarantine) and Continental Australia, Tasmania, and New Caledonia (all 7 days’ quarantine). All of these jurisdictions delayed the arrival of the pandemic by implementing their own full maritime quarantine (*2*–*7*) (Figure), although in the case of New Caledonia the quarantine was imposed by Australia. In each of these jurisdictions, local health officials credited the success in delaying influenza to strict maritime quarantine.

**Figure:**
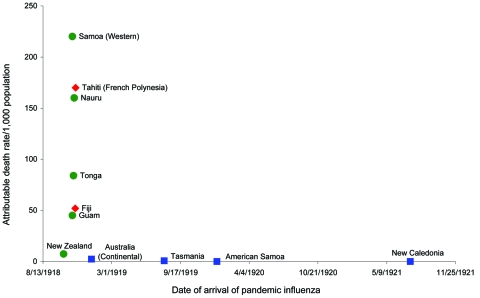
Comparison of attributable mortality rate from pandemic influenza versus time of arrival of influenza into South Pacific Island jurisdictions for the pandemic beginning in 1918. Sources for mortality data with wave-specific crude mortality rates per 1,000 population (r) from pandemic influenza: American Samoa (r = 0) (*7*,*8*); Australia (Continental) (r = 2.4) (*9*); Fiji (r = 52) (*2*); Guam (r = 45) (*8*,*10*); Nauru (r = 160) (*3*); New Caledonia (r<10) (*11*); New Zealand (r = 7.4) (*12*); Samoa (r = 220) (*2*); Tahiti (r = 190) (*13*); Tasmania (r = 0.81) (*6*); and Tonga (r = 840) (*2*). Sources for date of pandemic influenza arrival data (where different from the source of the mortality data detailed above): Australia (Continental) (*5*). Blue square, strict maritime quarantine; red diamond, incomplete maritime quarantine; green circle, no border control.

While it was in force, the maritime quarantine used by American Samoa from November 23, 1918, appeared to exclude pandemic influenza (*2*). Once influenza did reach this jurisdiction in 1920, no recorded deaths were attributed to influenza (in a population of ≈8,000) (*8*). In contrast, influenza spread rapidly through Western Samoa (now named Samoa). The impact was amplified by a lack of medical assistance and by food shortages in the area. Western Samoa had the worst death rate for any country or territory recorded in the 1918 pandemic, losing 19%–22% of its population (*2*).

Continental Australia implemented a maritime quarantine in October 1918. The arrival of influenza was delayed until early January 1919 (*14*), 3 months after the pandemic had appeared in New Zealand, where no systematic form of border control was in effect. The Australian island state of Tasmania instituted a strict maritime quarantine beginning January 27, 1919, once the Australian state of New South Wales had reported cases of pandemic influenza (*4*). Pandemic influenza did not penetrate into Tasmania until August 1919, and when it did, the chief health officer noted that it was a milder infection than experienced on mainland Australia. The resulting death rate for Tasmania of 0.81/1,000 population (*6*) was one of the lowest recorded worldwide.

New Caledonia was protected from the pandemic until 1921 by the strict 7-day quarantine of outbound vessels from Australian ports that began in late 1918 (*11*,*15*). Visiting ships from Sydney and Wallis Island were the eventual source of an influenza outbreak that began on July 17, 1921 (*11*).

Partial quarantine (as defined by the routine release, without quarantine, of asymptomatic passengers) proved unsuccessful in both Fiji and Tahiti in French Polynesia in 1918 (*2*,*16*). The other island jurisdictions that were identified as using no measures of border control (see circles in Figure) experienced the arrival of pandemic influenza at similar times.

The Figure also shows the death rates attributed to pandemic influenza per 1,000 total population compared with the date of the first recorded cases (for those jurisdictions for which date of first case and mortality data in the second wave of the 1918 pandemic were available). The jurisdictions of Australia, Tasmania, New Caledonia, and American Samoa appear to have benefited from a lower death rate resulting from delay in the arrival of influenza. Also, the lower death rates in some of these countries may have been partly attributable to such factors as preexisting levels of immunity, various socioeconomic characteristics of the populations (e.g., differing levels of poverty), and demographic factors (e.g., crowding and rurality). Unfortunately, limitations of available historical data prevented exploring these issues.

## Conclusions

Strict maritime quarantine appears to have been a successful method for delaying and excluding influenza for at least 4 South Pacific Island jurisdictions in the influenza pandemic that began in 1918. Some of these apparent benefits of maritime quarantine may have been attributable to minimal ship contact and geographic remoteness, but these explanations are unlikely given that there were ultimately few places protected in this way in the Pacific. The reasons for the lower mortality rates in jurisdictions that achieved successful delay are unclear. Viral attenuation over time is 1 possibility, although good supportive data for this and other explanations are lacking.

Nevertheless, the use of border control for the future protection of islands from pandemics must take into consideration the different nature of 21st-century societies, such as contact as a result of regular air travel. Island jurisdictions need to continue to undertake pandemic planning for effective border control (potentially with the assistance of larger nations or regional and international agencies). Because some of these jurisdictions involve widespread archipelagos, planning for within-country border control, especially for those populated islands with no airports, is also desirable.

Further modeling studies that are specific to the characteristics of island jurisdictions are also needed to better determine the probability that border control can succeed in the modern era. Nevertheless, now that influenza transmission is better understood, modifications could be made to enhance traditional border control measures to minimize disruptions. For example, in the event of a future pandemic, islands could potentially still trade by ship or plane if they did not allow crews to disembark and if they instituted effective infection control with ongoing surveillance of workers who handle cargo.

## References

[1] Uscher-Pines L, Omer SB, Barnett DJ, Burke TA, Balicer RD. Priority setting for pandemic influenza: an analysis of national preparedness plans. PLoS Med. 2006;3:e436. 10.1371/journal.pmed.0030436 17048982 PMC1609123

[2] Herda P. The 1918 influenza pandemic in Fiji, Tonga and the Samoas. In: Bryder L, Dow D, editors. New countries and old medicine: proceedings of an international conference on the history of medicine and health. Auckland, New Zealand: Pyramid Press, 1995.

[3] Tomkins S. The influenza epidemic of 1918–1919 in Western Samoa. J Pac Hist. 1992;27:181–97.

[4] Clarke A. Annual report 1918–1919. In: Department of Public Health. Hobart (Tasmania): Government Printer; 1919.

[5] McQueen H. “Spanish ’flu”–1919: political, medical and social aspects. Med J Aust. 1975;1:565–70. 1095898 10.5694/j.1326-5377.1975.tb111588.x

[6] Morris E. Annual report 1919–1920. In: Department of Public Health. Hobart (Tasmania): Government Printer; 1920.

[7] Ravenholt RT, Foege WH. 1918 influenza, encephalitis lethargica, parkinsonism. Lancet. 1982;320:860–4. 10.1016/S0140-6736(82)90820-0 6126720

[8] Crosby A. America’s forgotten pandemic: the influenza of 1918. Cambridge (UK): Cambridge University Press; 2003.

[9] Patterson KD, Pyle G. The geography and mortality of the 1918 influenza pandemic. Bull Hist Med. 1991;65:4–21. 2021692

[10] Underwood JH. Effects of the 1918 influenza pandemic mortality experience on subsequent fertility of the native population of Guam. Micronesica. 1984;19:1–10. 12267360

[11] Peltier F. L’épidémie d’influenza qui a sévi en Nouvelle Calédonie en 1921. Bulletin de l’Office International d'Hygiène Publique. 1922;14:676–85.

[12] Rice G. Black November: the 1918 influenza pandemic in New Zealand. Christchurch (New Zealand): Canterbury University Press; 2005.

[13] Allard M. L’épidémie d’influenza de 1918–1919 dans les colonies françaises. IV. - Colonies françaises de l’Océanie. AMedPhC. 1922;20:66–72.

[14] Holman W. Proclamation of N.S.W. Government regulations to control the epidemic. The Sydney Morning Herald. 1919 3 Feb.

[15] Berlioz-Arthaud A, Barr IG. Laboratory-based influenza surveillance in New Caledonia, 1999–2003. Trans R Soc Trop Med Hyg. 2005;99:290–300. 10.1016/j.trstmh.2004.07.004 15708388

[16] Reports on public health and medical subjects no. 4: report on the pandemic of influenza 1918–19. London: Her Majesty’s Stationary Office; 1920.

